# Modulation of Inflammatory Pathways and Adipogenesis by the Action of Gentisic Acid in RAW 264.7 and 3T3-L1 Cell Lines

**DOI:** 10.4014/jmb.2105.05004

**Published:** 2021-06-14

**Authors:** Min-jae Kang, Woosuk Choi, Seung Hyun Yoo, Soo-Wan Nam, Pyung-Gyun Shin, Keun Ki Kim, Gun-Do Kim

**Affiliations:** 1 Department of Microbiology, College of Natural Sciences, Pukyong National University, Busan 48513, Republic of Korea; 2 UCLA Children’s Discovery and Innovation Institute, Mattel Children’s Hospital UCLA, Department of Pediatrics, David Geffen School of Medicine, UCLA, Los Angeles, CA 90095, USA; 3 Biomedical Engineering and Biotechnology Major, Division of Applied Bioengineering, College of Engineering, Dong-Eui University, Busan 47340, Republic of Korea; 4 Himchan Agriculture Co., Ltd., Eumseong 27629, Republic of Korea; 5 Department of Life Sciences and Environmental Biochemistry, College of Natural Resources and Life Sciences, Pusan National University, Miryang 50463, Republic of Korea

**Keywords:** Gentisic acid, anti-inflammatory effect, anti-adipogenic effect, co-culture system

## Abstract

Gentisic acid (GA), a benzoic acid derivative present in various food ingredients, has been shown to have diverse pharmaceutical activities such as anti-carcinogenic, antioxidant, and hepatoprotective effects. In this study, we used a co-culture system to investigate the mechanisms of the anti-inflammatory and anti-adipogenic effects of GA on macrophages and adipocytes, respectively, as well as its effect on obesity-related chronic inflammation. We found that GA effectively suppressed lipopolysaccharide-stimulated inflammatory responses by controlling the production of nitric oxide and pro-inflammatory cytokines and modulating inflammation-related protein pathways. GA treatment also inhibited lipid accumulation in adipocytes by modulating the expression of major adipogenic transcription factors and their upstream protein pathways. Furthermore, in the macrophage–adipocyte co-culture system, GA decreased the production of obesity-related cytokines. These results indicate that GA possesses effective anti-inflammatory and anti-adipogenic activities and may be used in developing treatments for the management of obesity-related chronic inflammatory diseases.

## Introduction

Obesity is a condition characterized by an excessive accumulation of lipids in adipose tissue owing to various reasons, and it is regarded as a global public health issue [1]. Several studies have revealed that obesity is highly related to the occurrence of various diseases such as cancer, coronary heart disease, atherosclerosis, and hyperlipidemia, as well as type 2 diabetes mellitus [2-4]. In addition, recent research has shown that obesity is connected with the development of chronic low-grade inflammation which triggers insulin resistance and diabetes [5-7].

In obesity, hypertrophic adipocytes secrete tumor necrosis factor (TNF)-α, inducing the release of monocyte chemoattractant protein (MCP)-1 from preadipocytes [8]. TNF-α and MCP-1 can attract macrophages to adipose tissues, followed by the release of pro-inflammatory cytokines [6]. TNF-α additionally induces lipolysis in adipocytes leading to the production of free fatty acids (FFAs) [9], which then increases the oxidative stress in adipocytes and stimulates macrophage activation, resulting in the development of a paracrine loop of FFAs from adipocytes and TNF-α from macrophages. This paracrine loop enhances the adipocyte functions and immune regulation of macrophages [10]. Subsequently, this phenomenon promotes the development of insulin resistance and related metabolic disorders [11].

Gentisic acid (GA; 2,5-dihydroxybenzoic acid) is a benzoic acid derivative [12] found in various foods such as strawberries, apples [13], wheat [14], sesame [15], wine [16], and mushrooms [17,18]. GA is also a minor metabolite of aspirin, which is one of the most anti-inflammatory drugs available [19]. Several studies have revealed that GA has various pharmacological activities, including anti-inflammatory [20], anti-carcinogenic, and especially antioxidant effects [21]. However, the anti-adipogenic activity and effect of GA on obesity-related chronic inflammation have been poorly studied.

Therefore, in this study, we verified the anti-inflammatory activity of GA in RAW 264.7 macrophages and investigated the anti-adipogenic effects of GA in 3T3-L1 adipocytes. We additionally evaluated the inhibitory effects of GA on the production of pro-inflammatory molecules via the interaction of co-cultured RAW 264.7 cells and 3T3-L1 cells.

## Materials and Methods

### Reagents

GA, purchased from Sigma–Aldrich Inc. (Germany), was dissolved in dimethyl sulfoxide (DMSO; Sigma–Aldrich Inc.) and stored at 4°C for further use. Rabbit primary antibodies [inducible nitric oxide synthase, iNOS (cat. no. 13120); cyclooxygenase-2, COX-2 (cat. no. 12282); CCAAT/enhancer-binding protein alpha, C/EBPα (cat. no. 8178); peroxisome proliferator-activated receptor gamma, PPARγ (cat. no. 2435); nuclear factor kappa-light-chain-enhancer of activated B cells, NF-κB (cat. no. 8242); phosphorylated (p)-NF-κB (Ser 536; cat. no. 3033); p-IκB kinase, p-IKKα/β (Ser 176/180; cat. no. 2697); inhibitor of nuclear factor kappa B alpha, IκBα (cat. no. 9242); p-IκBα (Ser 32; cat. no. 2895); extracellular signal-regulated kinase, ERK1/2 (cat. no. 4695); p-ERK1/2 (Thr 202/Tyr 204; cat. no. 4370); p38 mitogen-activated protein kinase, p38MAPK (cat. no. 8690); p-p38MAPK (Thr 180/Tyr 182; cat. no. 4511); c-Jun N-terminal kinase, JNK1/2 (cat. no. 9252); p-JNK1/2 (Thr 183/Tyr 185; cat. no. 9255); adiponectin (cat. no. 2789); fatty acid synthase, FAS (cat. no. 3180); phosphatidylinositol 3-kinase, PI3K 110α (cat. no. 4249); mammalian target of rapamycin, mTOR (cat. no. 2983); p-mTOR (Ser 2481; cat. no. 2794); protein kinase B, Akt (cat. no. 9272); p-Akt (Ser 473; cat. no. 4060); β-catenin (cat. no. 8480); p-β-catenin (Ser 552; cat. no. 5651); p-β-catenin (Ser 33/37, Thr 41; cat. no. 9561); glycogen synthase kinase 3 beta, GSK3β (cat. no. 9315); p-GSK3β (Ser 9; cat. no. 5558); glyceraldehyde-3-phosphate dehydrogenase, GAPDH (cat. no. 5174)], IKKα mouse monoclonal antibody (cat. no. 11930), horseradish peroxidase (HRP)-conjugated anti-rabbit IgG secondary antibodies (cat. no. 7074), and HRP-conjugated anti-mouse IgG secondary antibodies (cat. no. 7076) were purchased from Cell Signaling Technology Inc. (USA). Anti-sterol regulatory element-binding transcription factor 1 (SREBP1) primary antibodies (cat. no. ab44153) were obtained from Abcam (UK).

### Cell Culture

RAW 264.7 murine macrophages and 3T3-L1 murine preadipocytes were purchased from the American Type Culture Collection (USA). RAW 264.7 cells were cultured in 10% heat-inactivated fetal bovine serum (FBS; USA) and 1% penicillin/streptomycin solution (PS; Corning) containing Dulbecco’s modified Eagle’s medium (DMEM; Corning). Additionally, 3T3-L1 cells were grown in DMEM supplemented with 10% fetal calf serum (CS; Gibco, USA) and 1% PS. Both cell lines were incubated at 37°C in a humidified atmosphere with 5% CO2, and the media were replaced every 2 days. Subculturing was performed when the cells attained 70% confluence.

### Cell Viability Assays

To evaluate the cytotoxic effect of GA on RAW 264.7 and 3T3-L1 cells, we performed the WST-1 assay [22]. RAW 264.7 cells (1 × 10^4^ cells/well) or 3T3-L1 cells (0.5 × 10^4^ cells/well) were seeded in 96-well plates and grown for 24 h for stabilization. After 24 h, the seeded cells were treated with various concentrations of GA (200, 400, 600, and 800 μM) for 24 h. Next, the medium was replaced with fresh medium containing 10% of EZ-Cytox Cell Viability Assay Solution (Daeil Lab Service, Korea) and incubated for an additional 2 h. The absorbance of the solution at 460 nm was measured using a VersaMax microplate reader (Molecular Devices, USA), which was used to calculate the cell viability.

### Nitric Oxide (NO) Production Assay

The inhibitory effect of GA on NO production in RAW 264.7 cells was evaluated using the Griess reagent (Sigma–Aldrich Inc.) [23]. RAW 264.7 cells were seeded into 24-well plates (5 × 10^4^ cells/well) and incubated for 24 h. After incubation, the cells were incubated in the absence and presence of GA (300 and 600 μM) for 2 h. Subsequently, except for the negative control, lipopolysaccharide derived from *Escherichia coli* O111:B4 (LPS; 1 μg/ml; Sigma–Aldrich Inc.) was added to each well and allowed to react for 22 h. After inducing inflammation using LPS, the supernatant of each well was collected and mixed with the same volume of Griess reagent. The reaction was performed in the dark, and the absorbance was measured at 540 nm using a VersaMax microplate reader (Molecular Devices).

### 3T3-L1 Preadipocyte Differentiation

To induce differentiation of 3T3-L1 preadipocytes into mature adipocytes, the cells were cultured until they attained 100% confluence. Next, the medium was replaced with fresh medium, and the cells were incubated for an additional 2 days. After 2 days (day 0), the cells were induced to differentiate in DMEM containing 10% FBS, 1%PS, and MDI [10 μg/ml insulin, 0.5 mM 3-isobutyl-1-methylxanthine (IBMX), and 1 μM dexamethasone (DEX)](Sigma–Aldrich Inc.) for the first 2 days. On days 2 and 4, the medium was replaced with DMEM supplemented with 10% FBS, 1% PS, and 1 μg/ml insulin. On day 6, the medium was replaced with DMEM containing 10% FBS and 1% PS, and the cells were incubated for two additional days.

### Oil Red O Staining

To investigate the inhibitory effects of GA on lipid accumulation during adipogenesis, 3T3-L1 cells were differentiated in 12-well plates with and without treatment with GA (300 and 600 μM) during every replacement of medium. After completing differentiation, the cells were rinsed with phosphate-buffered saline (PBS; Biosesang, Korea) and fixed with 0.4% formaldehyde solution (Junsei Chemical Co., Ltd., Japan; diluted in PBS) for 5 min. Cells were then washed with 60% isopropanol (Sigma–Aldrich Inc.) and fixed with 0.4% formaldehyde for 1 h. Formaldehyde was removed, and the cells were stained with 3.5 g/l of Oil Red O (Sigma–Aldrich Inc.; diluted in isopropanol) for 10 min. The stained cells were rinsed thrice with distilled water and observed using a phase-contrast microscope. To quantify the stained Oil Red O dye in lipid droplets, the dye was eluted using 100%isopropanol for 5 min. The absorbance of the eluted dye was measured at 540 nm using a VersaMax microplate reader (Molecular Devices).

### Coculture of 3T3-L1 and RAW 264.7 Cells

The secretion of inflammatory mediators from the coculture of 3T3-L1 and RAW 264.7 cells was measured and analyzed as described by Suganami *et al*. [10] with certain modifications. First, 3T3-L1 cells were seeded into 6-well plates, and differentiation was induced until day 8. On day 8, the medium was replaced with serum-free DMEM, and the cells were incubated for 24 h. After starvation, RAW 264.7 cells were added to plates containing starved and fully differentiated 3T3-L1 cells with and without treatment with GA (300 and 600 μM) and further incubated for 24 h. Subsequently, the supernatants were harvested to investigate the inflammatory cytokine production. As a control, equal numbers of 3T3-L1 cells were cultured separately to induce differentiation.

### Measurement of MCP-1 and TNF-α Secretion

Coculture supernatants were collected and the production of MCP-1 and TNF-α was analyzed using the MCP-1 Mouse ELISA (enzyme-linked immunosorbent assay) Kit and TNF-α Mouse ELISA Kit (Invitrogen, USA), according to the manufacturer’s instructions.

### Reverse Transcription-Polymerase Chain Reaction (RT-PCR)

Total RNA from 3T3-L1 and RAW 264.7 cells treated with and without GA was extracted using the RNeasy Plus Mini Kit (Qiagen, Germany) according to the manufacturer’s protocol. The concentration of extracted RNA was measured using a NanoDrop 2000 Spectrophotometer (Thermo Fisher Scientific, USA). cDNA was synthesized from 50 ng of each RNA sample using SuPrimeScript RT Premix (GeNet Bio, Korea). PCR was performed with equal amounts of synthesized cDNA using Prime Taq Premix (GeNet Bio) and specific primers (Table 1) under the same conditions for RNA samples of both cells (95°C for 1 min, 59°C for 1 min, and 72°C for 1 min; 30 cycles). The PCR products were stained using EcoDye (BioFACT, Korea) and visualized using the iBright CL1000 Imaging System (Invitrogen).

### Protein Extraction and Western Blotting Analysis

To analyze the effects of GA treatment on protein expression, the cells were rinsed with PBS and harvested in ice-cold PBS using a cell scraper. The collected cells were lysed using PRO-PREP Protein Extraction Solution (iNtRON Biotechnology, Korea) for 30 min at 4°C. The lysates were centrifuged for 20 min at 16,000 ×*g*, and the proteins in the supernatants were collected. Concentration of the proteins was determined using the Bradford reagent (Biosesang). For western blotting analysis, equal amounts of each protein were mixed with Laemmli sample buffer and boiled for 5 min. Proteins were separated on a 12% sodium dodecyl sulfate-polyacrylamide gel, and the resolved proteins were transferred to a nitrocellulose membrane (PALL Life Sciences, USA). The membranes were blocked with PBS and Tween 20 (PBST) containing 5% skim milk (BioShop, Canada) for 1 h and rinsed with PBST for 30 min. Following washing, the membranes were incubated with specific primary antibodies at 4°C overnight and washed with PBST for 15 min. After washing, the blots were incubated with HRP-conjugated secondary antibodies (Cell Signaling Technology Inc.) for 1 h at room temperature and rinsed with PBST for 30 min. The proteins bound to secondary antibodies were made to react with an enhanced chemiluminescence solution (AbFrontier, Korea), and the reaction was detected using an IBright Imaging System (Thermo Fisher Scientific).

### Statistical Analysis

Statistical analyses were performed using Prism 5.0 software (GraphPad Software, USA). One-way ANOVA and Dunnett’s multiple comparison test were used to analyze the differences between each experimental group. All data are presented as the means ± SD, and *p* < 0.05 is considered as statistically significant.

## Results

**Fig. 1:**
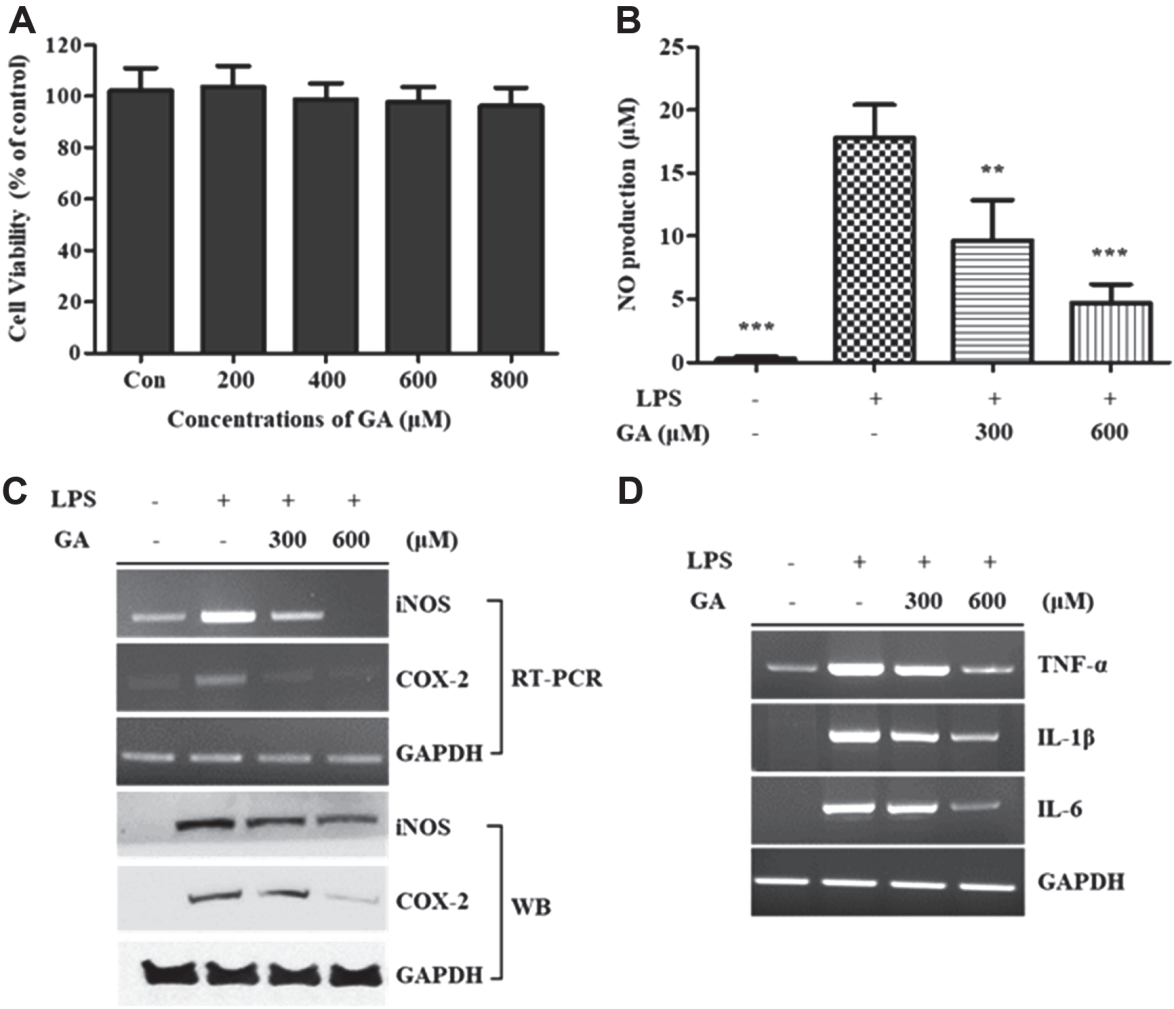
Effects of GA treatment on cell viability and inflammatory responses in RAW 264.7 cells. (**A**) RAW 264.7 cells were incubated with and without GA (200, 400, 600, and 800 μM) for 24 h. Cell viability was measured using WST-1 assays. (**B–D**) RAW 264.7 cells were pre-incubated with GA (300 and 600 μM) for 2 h and then stimulated using LPS treatment for 22 h. Supernatants were harvested, and the production of NO was determined using the Griess reagent. The mRNA and protein expression levels of iNOS and COX-2 from whole-cell lysates were measured using RT-PCR and WB, respectively. The mRNA levels of TNF-α, IL-1β, and IL-6 in whole cells were measured using RT-PCR. Data in the graph are presented as the mean ± SD of three independent experiments. **p < 0.01, ***p < 0.001 vs. the LPS-stimulated group. GAPDH was used as a loading control. GA, gentisic acid; LPS, lipopolysaccharide; iNOS, inducible nitric oxide synthase; COX-2, cyclooxygenase-2; TNF-α, tumor necrosis factor-α; IL, interleukin; GAPDH, glyceraldehyde 3-phosphate dehydrogenase; RT-PCR, reverse transcription-polymerase chain reaction; WB, western blotting.

To determine the cytotoxicity of GA to RAW 264.7 cells, we performed WST-1 assay using various concentrations of GA (200, 400, 600, and 800 μM) for the treatment of RAW 264.7 cells for 24 h. As shown in Fig. 1A, GA treatment did not show considerable cytotoxicity up to a concentration of 800 μM. We then determined the inhibitory effects of GA on NO production in LPS-stimulated RAW 264.7 cells. The results showed that GA treatment effectively prevented the secretion of NO dose-dependently compared to that in the LPS-stimulated group not treated with GA (Fig. 1B). RT-PCR and western blotting analyses were performed to investigate the expression of major inflammatory enzymes, such as iNOS and COX-2. Both the mRNA and protein expression levels of iNOS and COX-2 were suppressed via GA treatment compared to the LPS-stimulated group (Fig. 1C). GA treatment specifically inhibited the mRNA expression levels of pro-inflammatory cytokines, including TNF-α, interleukin (IL)-1β, and IL-6 (Fig. 1D).

### Effects of GA on Cell Viability and Adipogenic Response in 3T3-L1 Cells

**Fig. 2:**
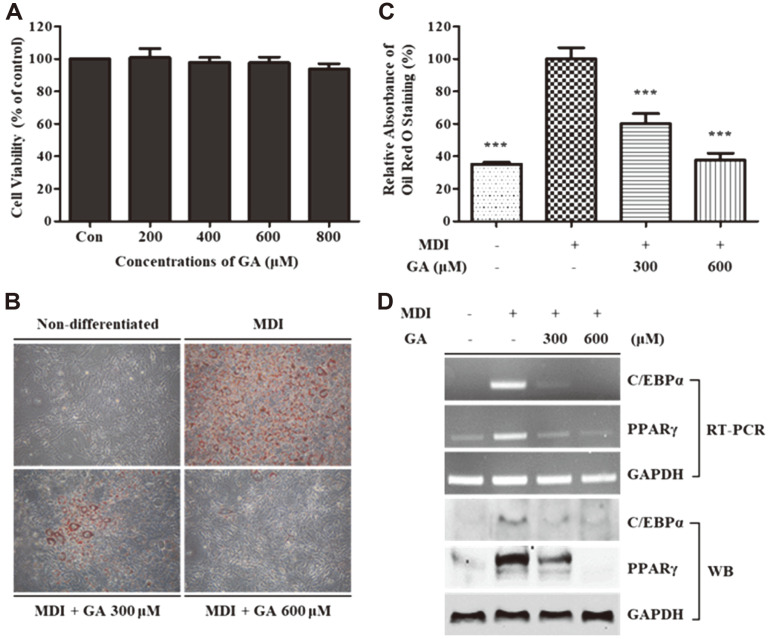
Effects of GA treatment on cell viability and adipogenic response in 3T3-L1 cells. (**A**) 3T3-L1 cells were incubated for 24 h in the presence or absence of GA (200, 400, 600, and 800 μM), and cell viability was determined using WST- 1 assay. (**B, C**) 3T3-L1 cells were induced to differentiate for 8 days in MDI-containing media with and without GA treatment (300 and 600 μM). At day 8, the lipid droplets in the cells were stained with Oil Red O and photographed using an invertedphase microscope at 100× magnification. After obtaining images, the retained dye was eluted and quantified using a microplate reader. Data in the graph are presented as the mean ± SD of three independent experiments. ***p < 0.001 vs. the fully differentiated group. (**D**) After differentiation, the cells were harvested and the mRNA and protein expression levels of C/EBPα and PPARγ were measured using RT-PCR and WB, respectively. GAPDH was used as a loading control. GA, gentisic acid; MDI, mixture of IBMX, DEX, and insulin; C/EBPα, CCAAT/enhancer-binding protein α; PPARγ, peroxisome proliferator-activated receptor γ; GAPDH, glyceraldehyde 3-phosphate dehydrogenase; RT-PCR, reverse transcription-polymerase chain reaction; WB, western blotting.

The WST-1 assay was performed to measure cell viability after GA treatment (200, 400, 600, and 800 μM) of 3T3-L1 cells for 24 h. GA treatment did not show considerable cytotoxic effects until a concentration of 800 μM on 3T3-L1 cells (Fig. 2A). To investigate the effects of GA on the adipogenesis in 3T3-L1 cells, we first executed Oil Red O staining. As shown in Figs. 2B and 2C, GA treatment successfully inhibited lipid accumulation in a dose-dependent manner in comparison to that in fully differentiated 3T3-L1 cells. The mRNA and protein expression of major adipogenic transcription factors, C/EBPα and PPARγ, were measured using RT-PCR and western blotting analysis. Results showed that GA treatment reduced the mRNA and protein expression of crucial adipogenesis-related transcription factors (Fig. 2D).

### Effects of GA on NF-κB and MAPK Signaling Pathways in LPS-Stimulated RAW 264.7 Cells

**Fig. 3:**
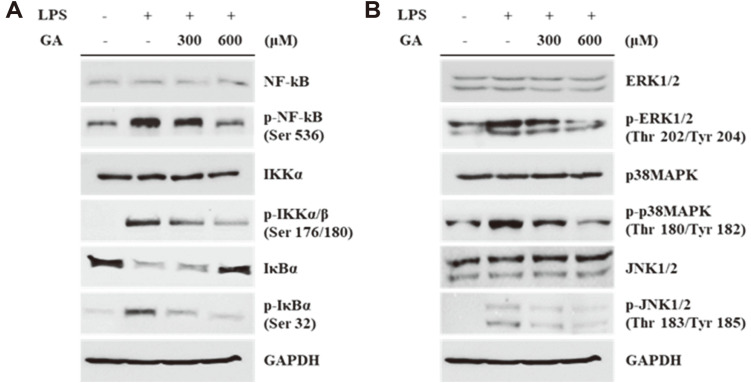
Effects of GA treatment on NF-κB and MAPK signaling pathway in LPS-stimulated RAW 264.7 cells. RAW 264.7 cells were pre-treated with GA (300 and 600 μM) for 2 h and then stimulated via LPS for 2 h. (**A**) The protein expression levels of total and phosphorylated forms of NF-κB, IKKα/β, and IκBα were determined using western blotting. (**B**) Western blotting was performed for detecting the protein expression of MAPKs and their phosphorylated forms. GAPDH was used as a loading control. GA, gentisic acid; LPS, lipopolysaccharide; NF-κB, nuclear factor kappa-light-chain-enhancer of activated B cells; IKK, IκB kinase; IκBα, inhibitor of nuclear factor kappa B-α; ERK, extracellular signal-regulated kinase; MAPK, mitogen-activated protein kinase; JNK, c-Jun N-terminal kinase; GAPDH, glyceraldehyde 3-phosphate dehydrogenase.

To investigate the proteomic effects of GA treatment on inflammatory responses, the protein expression of NF-κB pathway-related proteins and MAPKs was analyzed using western blotting. As shown in Fig. 3A, GA treatment inhibited the LPS-induced phosphorylation of NF-κB, IKKα/β, and IκBα. Total expressions of NF-κB and IKKα did not change markedly; however, IκBα expression was upregulated in the GA-treated group. Additionally, the phosphorylation of MAPKs (ERK, p38MAPK, and JNK) induced via LPS stimulation was downregulated via GA treatment. However, the expression of total MAPKs did not change considerably in any of the experimental groups (Fig. 3B).

### Effects of GA on Adipogenesis-Related Protein Expression in 3T3-L1 Cells

**Fig. 4:**
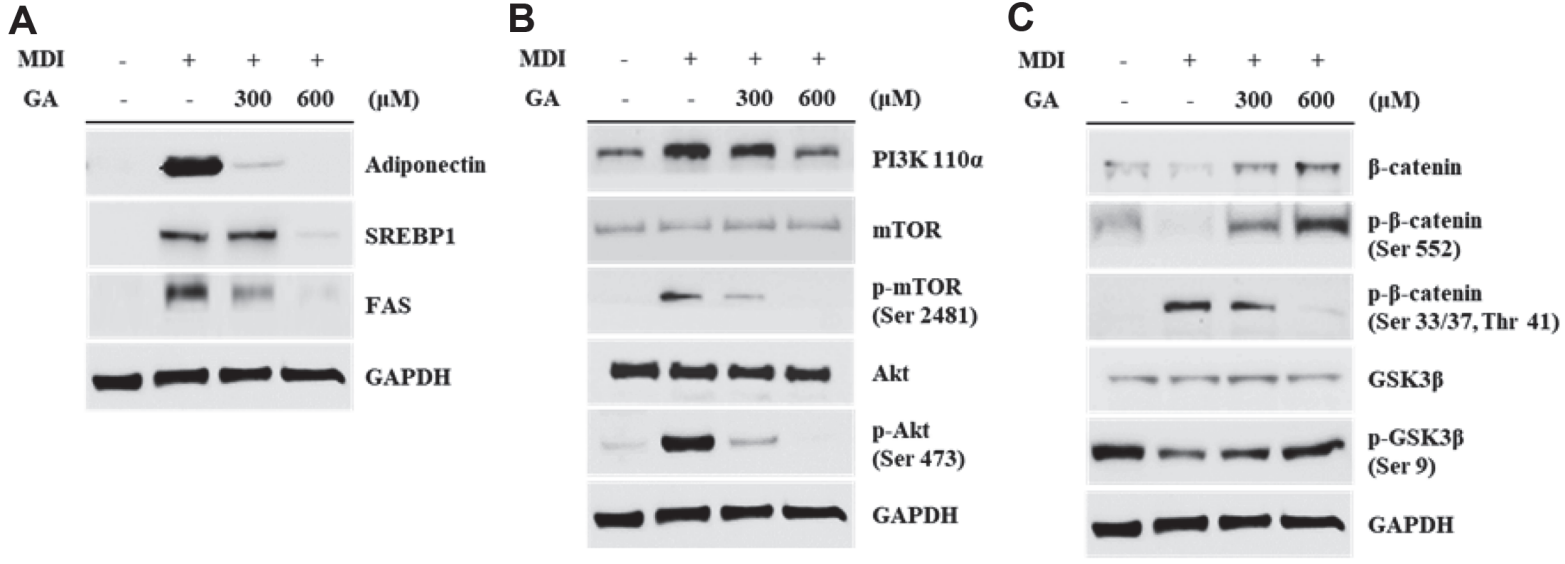
Effects of GA treatment on the adipogenesis-related protein expression in 3T3-L1 cells. Western blotting was performed to analyze the expression of adipogenic proteins. After 8 days of inducing differentiation with or without GA treatment (300 and 600 μM), the cells were harvested and the proteins were extracted for analysis. (**A**) Protein expression levels of adiponectin, SREBP1, and FAS. (**B**) Protein expression and phosphorylation levels of PI3K pathway proteins (PI3K 110α, mTOR, and Akt). (**C**) Protein expression levels of β-catenin, GSK3β, and their phosphorylated forms. GAPDH is used as a loading control. GA, gentisic acid; MDI, mixture of IBMX, DEX, and insulin; SREBP1, sterol regulatory element-binding protein 1; FAS, fatty acid synthase; PI3K, phosphatidylinositol 3-kinase; mTOR, mammalian target of rapamycin; Akt, protein kinase B; GSK3β, glycogen synthase kinase 3β; GAPDH, glyceraldehyde 3-phosphate dehydrogenase.

Western blotting was performed to study the anti-adipogenic effects of GA on protein expression levels in 3T3-L1 cells. As shown in Fig. 4A, the level of adiponectin, an adipogenesis marker, decreased in the GA-treated group. Additionally, the expression of lipid synthesis-related proteins, SREBP1 and FAS, was inhibited via GA treatment as compared to that in fully differentiated 3T3-L1 cells. Moreover, the protein expression of PI3K was suppressed in the GA-treated group, and phosphorylation of the PI3K downstream proteins including mTOR and Akt, was suppressed (Fig. 4B). In the β-catenin and GSK3β signaling, the protein expression levels of total β-catenin and GSK3β were not affected after GA treatment. The active form of β-catenin (phosphorylated at serine 552 residue) was increased via GA treatment, whereas GA treatment inhibited the phosphorylation of β-catenin at serine 33 and 37 and threonine 41 residues. In addition, phosphorylation of GSK3β was increased via GA treatment (Fig. 4C).

### Effects of GA on Cytokine Secretion in Macrophage-Adipocyte Coculture System

**Fig. 5:**
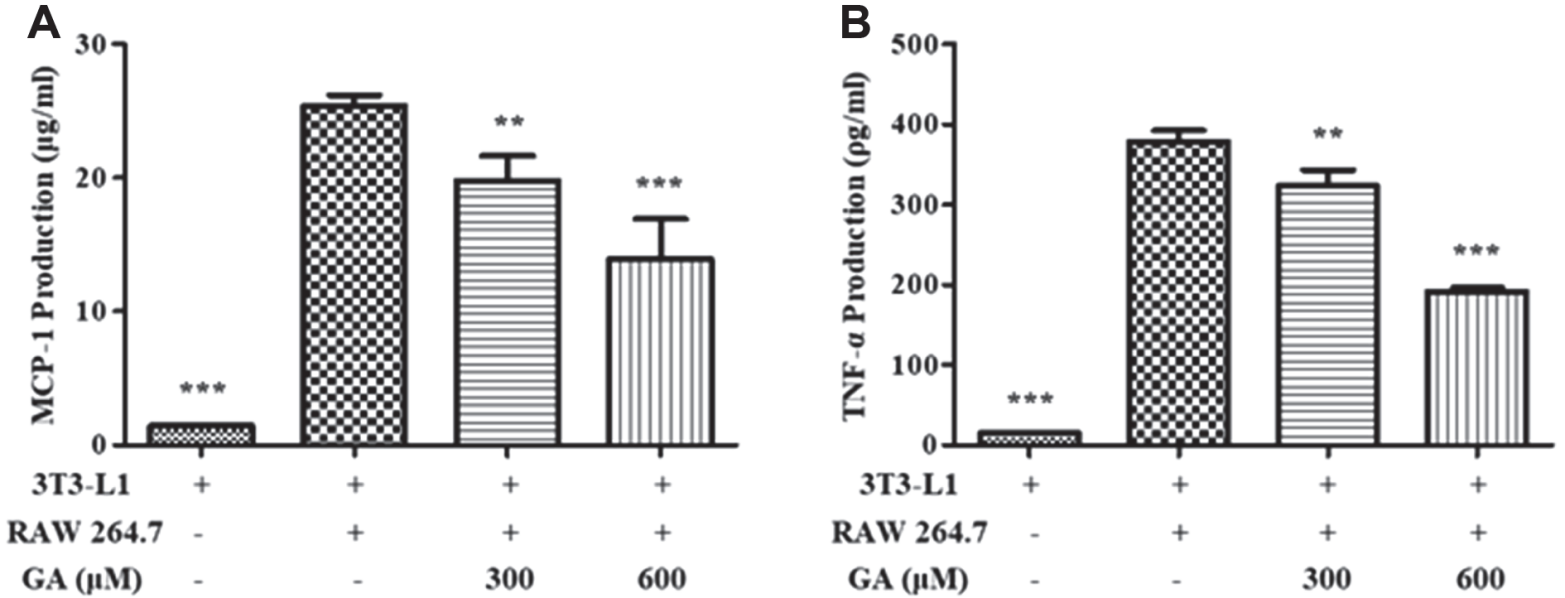
Effects of GA treatment on cytokine secretion in a macrophage–adipocyte coculture system. Starved and fully differentiated 3T3-L1 cells were cocultured with RAW 264.7 cells in a direct-contact system in the presence or absence of GA (300 and 600 μM) for 24 h. Supernatants were collected and analyzed using ELISA for measuring the production levels of (**A**) MCP-1 and (**B**) TNF-α. Values are presented as mean ± SD of three independent experiments. **p < 0.01, ***p < 0.001 vs. coculture without GA treatment. GA, gentisic acid; MCP-1, monocyte chemoattractant protein-1; TNF-α, tumor necrosis factor-α; ELISA, enzyme-linked immunosorbent assay.

To evaluate the effects of GA treatment on the expression of key cytokines in an obesity-related chronic inflammation environment, we performed ELISA using supernatant of RAW 264.7 and 3T3-L1 direct cocultures. As shown in Fig. 5A, MCP-1 production was reduced in the GA-treated group compared to that in the coculture group without GA treatment. In addition, the secretion of TNF-α decreased via GA treatment (Fig. 5B).

## Discussion

The infiltration of macrophages into adipose tissues in patients with obesity can be lead to chronic inflammation and the development of obesity-related diseases [24]. Therefore, numerous studies have been performed to identify anti-inflammatory and anti-adipogenic compounds that inhibit obesity-related chronic inflammation. Our goal in this study was to examine the anti-inflammatory and anti-adipogenic activity of GA and investigate the suppressive effect of GA on obesity-related inflammatory cytokine secretion in an adipocyte–macrophage coculture system. First, to determine the cytotoxicity of GA to RAW 264.7 macrophages and 3T3-L1 preadipocytes, we performed WST-1 assays. As shown in Figs. 1A and 2A, GA did not exert cytotoxic effects on either macrophages or adipocytes at concentrations up to 600 μM. Therefore, 300 and 600 μM of GA were used to investigate the effects of GA on biological activities.

Regarding the anti-inflammatory activity of GA, we first measured the NO production after treating LPS-stimulated RAW 264.7 cells with GA. NO is one of the free radical molecules, and overexpressed NO can be trigger inflammation, tissue damage, and the secretion of pro-inflammatory cytokines; thus, it is used as a marker for inflammatory response [25]. NO production after LPS stimulation in RAW 264.7 cells was significantly inhibited via GA treatment, suggesting that GA shows anti-inflammatory activity (Fig. 1B). Moreover, the mRNA and protein expression levels of major inflammation-related enzymes iNOS and COX-2 were investigated using RT-PCR and western blotting, respectively. iNOS catalyzes the formation of NO from L-arginine, which plays a crucial role in the immune response; however, excess iNOS can cause inflammation and the development of related diseases [26]. Similarly, COX-2 is involved in inflammatory responses and induces the alteration of arachidonic acid to prostaglandin E2 (PGE_2_), a lipid of the prostanoid family that plays vital roles in inflammatory signaling cascades [27]. Hence, the downregulation of iNOS and COX-2 is expected to be an efficient strategy for suppressing inflammation. As shown in Fig. 1C, compared to the LPS-stimulated group, GA treatment affected the mRNA and protein expression of iNOS and COX-2. Additionally, the mRNA expression of pro-inflammatory cytokines, such as TNF-α, IL-1β, and IL-6, was measured using RT-PCR. The levels of these cytokines are elevated in inflammatory diseases and they play essential roles in the inflammation process [28-30]. GA treatment decreased the mRNA expression of TNF-α, IL-1β, and IL-6 in LPS-treated RAW 264.7 cells (Fig. 1D). Consequently, these results indicated that GA successfully suppressed the inflammatory responses induced via LPS stimulation in RAW 264.7 cells.

Additionally, to investigate the mechanism of the anti-inflammatory activity of GA, we measured the protein expression levels of NF-κB and MAPK pathway-associated proteins using western blotting analysis. NF-κB is a transcription factor that plays a pivotal role in inflammatory responses by inducing the expression of pro-inflammatory proteins including iNOS, COX-2, and cytokines, after exposure to stimuli such as LPS [31]. In the inert state, NF-κB is inactivated by the inhibition of nuclear translocation upon binding to its inhibitor IκB. When the cells are exposed to various inflammatory stimuli, IKKs are phosphorylated and promote the phosphorylation of IκB. Phosphorylated IκB induces ubiquitin-dependent degradation, and NF-κB can be phosphorylated and translocated into the nucleus to initiate the transcription of pro-inflammatory genes [32, 33]. MAPKs include ERK, p38, and JNK; these proteins participate in various biological activities such as cell survival, proliferation, migration, and inflammation [34, 35]. During an inflammatory response, MAPKs can regulate the production of pro-inflammatory cytokines and enzymes. One of the downstream proteins of the MAPK cascade is NF-κB; activation of MAPKs can induce the transcriptional activity of NF-κB [36]. In this study, we demonstrated that GA inhibited the phosphorylation of NF-κB by inducing the expression of IκB and the suppression of IκBα degradation by restricting IKK phosphorylation. Therefore, the nuclear localization activity of NF-κB was inhibited (Fig. 3A). Additionally, phosphorylation of MAPKs, including ERK, p38, and JNK, was suppressed via GA treatment compared to that in cells treated with only LPS (Fig. 3B). These results suggest that GA treatment hindered NF-κB activation by inhibiting the phosphorylation of IκBα, IKK, and each MAPK protein, and subsequently, the production of pro-inflammatory enzymes and cytokines generated by NF-κB was attenuated.

After demonstrating its anti-inflammatory capacity, we evaluated the anti-adipogenic activity of GA. First, we investigated the inhibitory effect of GA on lipid accumulation in 3T3-L1 during adipogenesis by perfoming Oil Red O staining. As shown in Fig. 2B, GA treatment effectively suppressed the production of lipid droplets compared to that in the fully differentiated cell group when visualized under a microscope. The quantified results showed that GA treatment inhibited lipid accumulation by 60.2% and 37.7% at concentrations of 300 and 600 μM compared to the lipid content of the positive control, respectively (Fig. 2C). To further study the mechanism of the anti-adipogenic activity of GA, we analyzed the mRNA and protein expression levels of major adipogenesis-related factors, such as C/EBPα and PPARγ, using RT-PCR and western blotting analysis. C/EBPα and PPARγ are crucial transcription factors in the adipogenesis process; they promote their own expression by generating a positive feedback loop. These factors induce the transcription of important adipogenesis-related genes [37]. As shown in Fig. 2D, GA treatment inhibited the mRNA and protein expression of C/EBPα and PPARγ compared to that in fully differentiated cells. These results showed that GA treatment considerably suppressed lipid accumulation induced via adipogenesis by suppressing the C/EBPα and PPARγ expression.

To further study the inhibitory activity of GA on the adipogenic process, we measured the protein expression levels of adipogenesis-related factors. First, we investigated the protein expression levels of crucial adipogenic factors, such as adiponectin, SREBP1, and FAS. Adiponectin is produced in mature adipocytes and controls lipid metabolism; thus it can be considered as a marker of adipogenesis [38]. SREBP1 is a transcription factor that plays a pivotal role in adipogenesis in lipid accumulation by modulating the expression of adipogenic factors including FAS and PPARγ [39]. FAS promotes the enzymatic conversion of acetyl-CoA and malonyl-CoA to palmitate and is used as a substrate of long-chain fatty acids [40]. As shown in Fig. 4A, GA treatment suppressed the expression of adiponectin, SREBP1, and FAS. These results indicated that GA could inhibit not only C/EBPα and PPARγ but also the crucial factors involved in lipid synthesis processes, such as adiponectin, SREBP1, and FAS.

Next, we observed the expression patterns of proteins upstream of the adipogenesis process, such as the proteins connected with the PI3K and β-catenin pathways. The PI3K pathway, which is characterized by the activity of Akt and mTOR, is known to be involved in the adipogenic process and promotes the expression of adipogenesis-related factors. Specifically, PI3K is activated by forming a complex with the phosphorylated insulin receptor substrate. Subsequently, the activated PI3K serves as a phosphate group for Akt, and the phosphorylated Akt enhances the expression of PPARγ and supports glucose uptake [41]. mTOR is a target of Akt and it has been reported that phosphorylated mTOR can regulate adipogenesis and lipogenesis by promoting the expression of PPARγ and SREBP1 [42]. We found that GA effectively suppressed the activation of the PI3K-Akt-mTOR pathway (Fig. 4B). These results indicate that GA may regulate lipogenesis and adipogenesis by controlling the upstream pathway proteins of PPARγ and SREBP1.

We measured the expression levels of β-catenin signaling proteins including β-catenin and GSK3β. In contrast to the previously mentioned proteins, β-catenin is known to suppress adipogenesis via inhibition of C/EBPα and PPARγ [43]. β-Catenin is activated via the phosphorylation of its serine 552 residue. However, phosphorylation of its serine 33 and 37 and threonine 41 residues leads to the ubiquitination-mediated degradation of β-catenin. This degradation process is induced by GSK3β; however GSKβ is inactivated when its serine 9 residue is phosphorylated [44]. As shown in Fig. 4C, the protein expression levels of the total form and the form phosphorylated at serine 552 residue β-catenin were increased via GA treatment. In contrast, the phosphorylated form of β-catenin at serine 33 and 37 and threonine 41 was decreased in GA-treated cells. Moreover, phosphorylation at the serine 9 residue of GSK3β was inhibited in the GA-treated group. These results indicated that GA treatment might have prevented the degradation of β-catenin via inactivation of GSK3β; therefore, β-catenin could suppress the induction of C/EBPα and PPARγ.

After we verified the anti-adipogenic and anti-inflammatory activities of GA, we investigated the inhibitory effect of GA on cytokine production in obesity-related inflammation. To stimulate macrophage infiltration in obesity-associated tissues, we developed a coculture system. In obese adipose tissues, adipocytes secrete MCP-1 and FFAs. The released FFAs promote macrophage infiltration and recruitment, and the macrophages produce pro-inflammatory cytokines such as TNF-α. TNF-α stimulates the lipolysis followed by the secretion of FFAs, which comprise a loop of MCP-1 and TNF-α production. This phenomenon can cause obesity-related chronic inflammation and the development of associated diseases [45]. The production of MCP-1 and TNF-α induced by coculturing adipocytes and macrophages was suppressed via GA treatment (Fig. 5). Based on these results, we demonstrated that GA inhibited various obesity-related inflammatory responses.

In conclusion, we first investigated the anti-adipogenic and anti-inflammatory activities of GA and further examined the effect of GA treatment on obesity-related inflammatory responses. We found that GA effectively suppressed inflammation stimulated via LPS treatment by regulating the expression of major pro-inflammatory enzymes and cytokines, as well as regulating the expression of NF-κB and MAPK pathway-associated proteins in RAW 264.7 macrophages. In 3T3-L1 adipocytes, GA treatment considerably inhibited lipid accumulation during adipogenesis by regulating the expression of crucial adipogenic transcription factors and upstream pathway proteins, including PI3K-Akt-mTOR and β-catenin. Additionally, in the adipocyte-macrophage coculture system, the production of MCP-1 and TNF-α induced via the cross-talk of the two cells was suppressed after GA treatment. The current study revealed that GA has potential in developing novel agents for the management of obesity-related inflammatory diseases.

## Figures and Tables

**Table 1:** Primers for reverse-transcription polymerase chain reaction.

Genes	Forward primers	Reverse primers
*Nos2*	AAGCACATGCAGAATGAGTACCG	GTGGGACAGCTTCTGGTCGAT
*Cox2*	GCACTACATCCTGACCCACT	CCCAGGTCCTCGCTTATGAT
*Tnf*	CCCCTCAGCAAACCACCAAGT	CTTGGGCAGATTGACCTCAGC
*Il1b*	AATCTCACAGCAGCACATCAA	AGCCCATACTTTAGGAAGACA
*Il6*	GGAGGCTTAATTACACATGTT	TGATTTCAAGATGAATTGGAT
*Cebpa*	TGGACAAGAACAGCAACGAGT	GCGGTCATTGTCACTGGTCA
*Pparg*	GGTGCCAGTTTCGATCCGTA	GGTCATGAATCCTTGGCCCT
*Gapdh*	GAAGGTCGGTGTGAACGGAT	ACTGTGCCGTTGAATTTGCC

*Nos2*, inducible nitric oxide synthase; *Cox2*, cyclooxygenase-2; *Tnf*, tumor necrosis factor-α; *Il1b*, interleukin 1 beta; *Il6*, interleukin 6; *Cebpa*, CCAAT/enhancer-binding protein α; *Pparg*, peroxisome proliferator-activated receptor γ; *Gapdh*, glyceraldehyde 3-phosphate dehydrogenase
